# Supplementary material for: Elucidating the Anti-Tumorigenic Efficacy of Oltipraz, a Dithiolethione, in Glioblastoma
Source: Cells. 2022 Sep 29;11(19):3057. doi: 10.3390/cells11193057 (PMC9562012; doi:10.3390/cells11193057)
Supplement: Supplementary file 1 [file cells-11-03057-s001.zip › cells-1799615-supplementary.pdf]

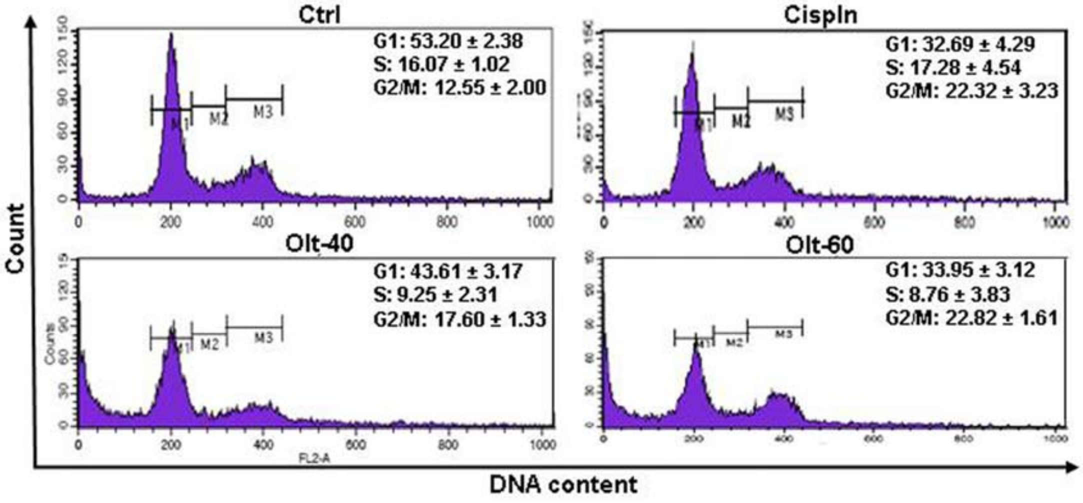

Figure S1: Representative image showing cell cycle pattern of U-87 MG cells following exposure of Cispln and Olt (40- and 60μM) for 48 h.

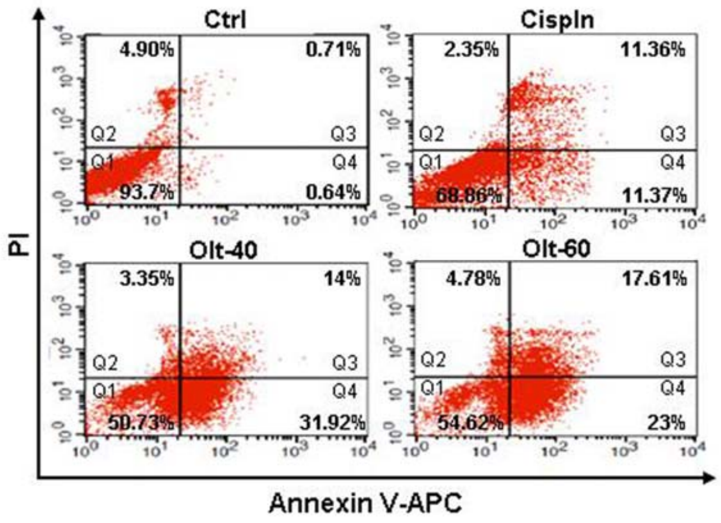

Figure S2: Representative image showing the annexin-PI pattern of U-87 MG cells following exposure of Cispln (30 μM) and Olt (40- and 60μM) for 48 h.

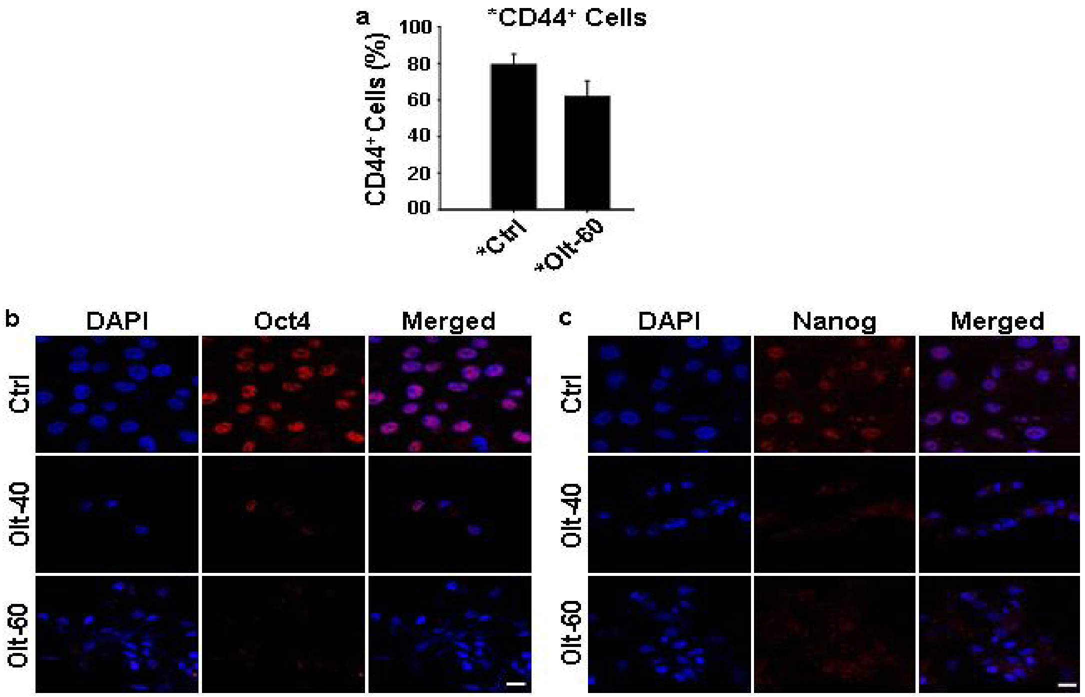

Figure S3: (a). Decrease in $CD44^+$ population in post-sorted $CD44^+$ cells ($*CD44^+$) exposed to Olt-60μM for 48 h. $*p \leq 0.05$, compared to the untreated $*CD44^+$ Ctrl group. Data are presented as mean ± SEM (n=4). **(**b,c). Immunostaining for detection Oct4 (b) and Nanog (c) in U-87 MG cells after treatment of Olt for 48 h suggested a decrease in expression of stem cell markers in response to Olt exposure.

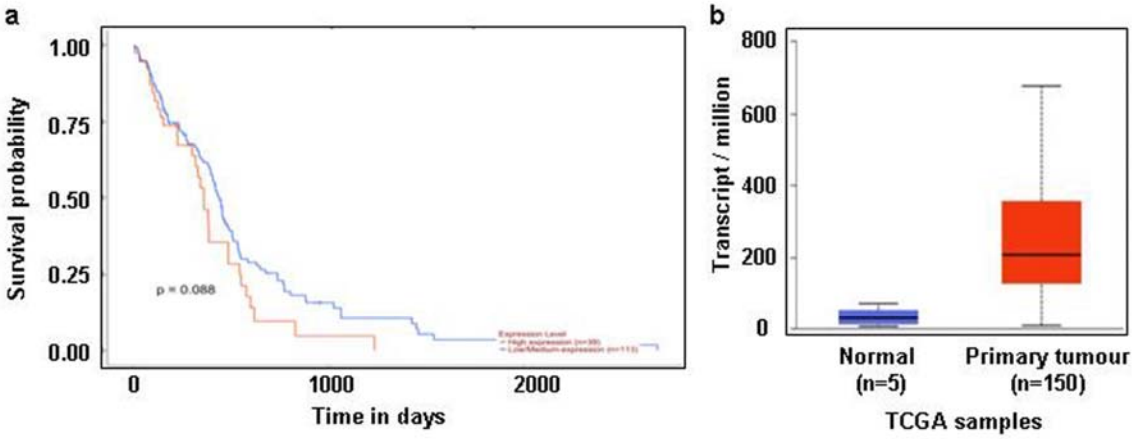

Figure S4: (a) *In silico* analysis with the data from The Cancer Genome Atlas (TCGA) for the relative CD44 expression in normal (n=5) and primary tumor (n=156) samples using the ULCAN web. (b) The survival rates of patients with GBM having low (blue) or high (red) CD44 expression, as estimated by the Kaplan-Meier method.
